# Epidemic Trends in High Tuberculosis Burden Countries During the Last Three Decades and Feasibility of Achieving the Global Targets at the Country Level

**DOI:** 10.3389/fmed.2022.798465

**Published:** 2022-03-03

**Authors:** Cheng Ding, Ming Hu, Yanwan Shangguan, Wanru Guo, Shuting Wang, Xuewen Feng, Zunjing Zhang, Ying Zhang, Kaijin Xu

**Affiliations:** ^1^State Key Laboratory for Diagnosis and Treatment of Infectious Diseases, National Clinical Research Center for Infectious Diseases, National Medical Center for Infectious Diseases, Collaborative Innovation Center for Diagnosis and Treatment of Infectious Diseases, The First Affiliated Hospital, Zhejiang University School of Medicine, Hangzhou, China; ^2^Lishui Hospital of TCM, Lishui, China

**Keywords:** tuberculosis, Burden of Disease, epidemics, forecasting, public health surveillance

## Abstract

**Objective:**

To estimate the epidemic trends of tuberculosis (TB) in 30 high burden countries (HBCs) over the past 30 years, which is crucial for tracking the status of disease control, especially at the country level.

**Methods:**

Annual data on incidence and mortality of TB in these 30 HBCs were extracted from the Global Burden of Disease database. The average annual percent change (AAPC) was used to evaluate the trends of incidence and mortality. The trajectory analysis was used to identify different trends among the subgroup countries. The predicted incidence and mortality rates in 2025, 2030, and 2035 were also calculated.

**Results:**

The incidence and mortality decreased in most of the HBCs. The AAPCs of incidence ranged between −4.0 (Indonesia) and −0.2% (DR Congo) (all *p* < 0.05). The incidence trends in Lesotho (AAPC: 0%, 95% *CI*: −0.4, 0.3, *p* = 0.8) and South Africa (AAPC: −0.2%, 95% *CI*: −0.5, 0, *p* = 0.1) were stable, and increased in Kenya with AAPC of 0.1% (95% *CI*: 0.1, 0.2, *p* < 0.05). The AAPCs for mortality ranged between −5.8 (Ethiopia) and −0.6% (Central African Republic) (all *p* < 0.05). The mortality trends in DPR Korea (AAPC: 0.1%, 95% *CI*: −0.3, 0.4, *p* = 0.6) and Russian Federation (AAPC: −0.5%, 95% *CI*: −1.9, 0.9, *p* = 0.5) were stable, and increased in Lesotho and Zimbabwe with AAPC of 1.3% (95% *CI*: 1.1, 1.4, *p* < 0.05) and 1.6% (95% *CI*: 1.0, 2.2, *p* < 0.05), respectively. Trajectory analysis showed that the Central African Republic, Lesotho, Cambodia, Namibia, and South Africa had higher incidences, and the Central African Republic had higher mortality. Brazil and China had relatively lower rates of incidence and mortality. Predictions showed that reduction rates of incidence and mortality could hardly be reached compared with those set for the global targets for the majority HBCs.

**Conclusions:**

The disease burden of TB has been reduced among the majority HBCs over the last three decades. According to the current control levels, achieving the ambitious global targets at the country level for these 30 HBCs is challenging.

## Introduction

Tuberculosis (TB) is caused by *Mycobacterium tuberculosis* and has affected humans for thousands of years (1) as the disease has occurred in every country around the world. Although TB can be completely cured with the proper treatment regimen, it remains one of the top 10 causes and the leading cause of death from a single infectious agent in 2019 (2) and the second only behind COVID-19 in 2020 (1.5 million died due to TB) (3). Although a reduction was observed in the absolute number of deaths from 2.38 million in 2000 to 1.41 million in 2019, the number of cases declined slowly in both absolute terms and per capita in recent years (2).

Global Burden of Disease (GBD) studies illustrated a heavy global burden of TB with 9.02 million incident cases and 1.21 million deaths in 2016 (4). Among all diseases judged by prevalent cases, TB ranked the third leading cause in 1990 and became the fourth in 2017 (5). Burden of TB varies greatly among countries and territories. Geographically, most TB cases were in the WHO regions of South-East Asia (44%), Africa (25%), and the Western Pacific (18%) in 2019. Particularly, the top 20 countries with the largest absolute numbers of estimated incident TB cases accounted for 84% of global incident cases (2). To provide a focus for global action, the 20 countries plus the top 10 countries with the highest estimated TB incidence rate are currently listed as the 30 high burden countries (HBCs) (2).

Global efforts to reduce the disease burden were focused on achieving targets within the context of the Millennium Development Goals (6), which were succeeded by the Sustainable Development Goals (7), and the post-2015 global TB strategy—the End TB Strategy—for the period 2016−2035 (8, 9). The main milestones and targets are quantified as 75% reduction in deaths and 50% reduction in the incidence (or <55 per 100,000) in 2025, and 90 and 80% (or <20 per 100,000) in 2030, and 95 and 90% (or <10 per 100,000) in 2035, compared with levels of 2015, respectively (8).

Evaluating epidemic trends and remaining challenges is crucial to tracking the success of TB prevention and control. The epidemics in these HBCs have a huge impact on achieving the goal of ending TB. However, currently, there is limited information on this issue. This study aims to assess the long-term epidemic trends among these HBCs, and additionally to provide unique insights on the feasibility of achieving the global targets at the country level.

## Materials and Methods

### Data Sources

Annual data on TB among the 30 HBCs from 1990 to 2019 were extracted by the Global Health Data Exchange (GHDx) query tool (10). Countries in the HBCs are: Angola, Bangladesh, Brazil, Cambodia, Central African Republic, China, Congo, Democratic People's Republic of Korea (DPR Korea), the Democratic Republic of the Congo (DR Congo), Ethiopia, India, Indonesia, Kenya, Lesotho, Liberia, Mozambique, Myanmar, Namibia, Nigeria, Pakistan, Papua New Guinea, Philippines, Russian Federation, Sierra Leone, South Africa, Thailand, United Republic of Tanzania (UR Tanzania), Vietnam, Zambia, and Zimbabwe (2).

The GHDx is a data catalog created and supported by the Institute for Health Metrics and Evaluation (IHME), which captures the health related data to populate the information for more than 350 diseases and injuries in 195 countries worldwide since 1990. The flexible design of the GBD makes it available for regular updates with new data and epidemiological studies involved (11). Protocols, data resources, definitions, efforts to improve the quality, and modeling were described in detail elsewhere (5, 12–14). Ethical approval was not required since the data included in this study were de-identified and publicly available.

### Statistical Analysis

The incidence and mortality were used as the main indicators, and age-standardized rates (ASRs) of which were adjusted by the world standard population (15). The average annual percent change (AAPC, %) and its 95% *CI* were estimated. The natural logarithm of rate was assumed to be linear along with time; that was Y = α + βX + ε, where Y referred to ln (rate), X represented the calendar year, and ε was the error, β represented the trend of the period segment for rate. AAPC was calculated as [(Exp (β) – 1)] × 100, and its 95% *CI* was obtained from the linear model. The *Z*-test was used to assess whether an AAPC value was different from zero. Subgroup trend analyses, such as age groups (1–14, 15–59, and ≥60 years) and sexes were involved. The mortality to incidence (MI) ratio was used as an indicator of disease severity as well as the capability level of clinical treatment and management strategies. The mean socio-demographic index (SDI) during 1990 and 2019 was also calculated, which was a composite indicator based on income, education, and fertility (5, 16).

To estimate a discrete mixture model for clustering of longitudinal data, the trajectory analysis with a censored normal model was conducted to identify distinct epidemic trends of TB and to classify countries into different subgroups. The Bayesian Information Criterion (BIC) log Bayes factor was used as the degree of evidence favoring the alternative model to classify the subgroups of countries. Value with more than 6 of BIC log Bayes factor indicated strong evidence to favor the alternative (more complex) model (17). Based on the AAPCs and the predicted linear slopes from the trajectory models, the TB incidence and mortality rates for the HBCs regarding the milestones (2025, 2030) and targets (2035) at the country level were predicted.

Average annual percent changes were calculated by the Joinpoint Regression Program (Version 4.9.0.0. March 2021). The trajectory model was conducted by Proc Traj with SAS 9.4 (SAS Institute Inc., Cary, NC, USA) (18). Origin 2019 (Microcal Software Inc., Northampton, MA, USA) and SAS 9.4 were used to draw the figures. The statistical significance level was set at 0.05 (two-side).

## Results

### Epidemic Trends of TB in the HBCs

**Figure 1:**
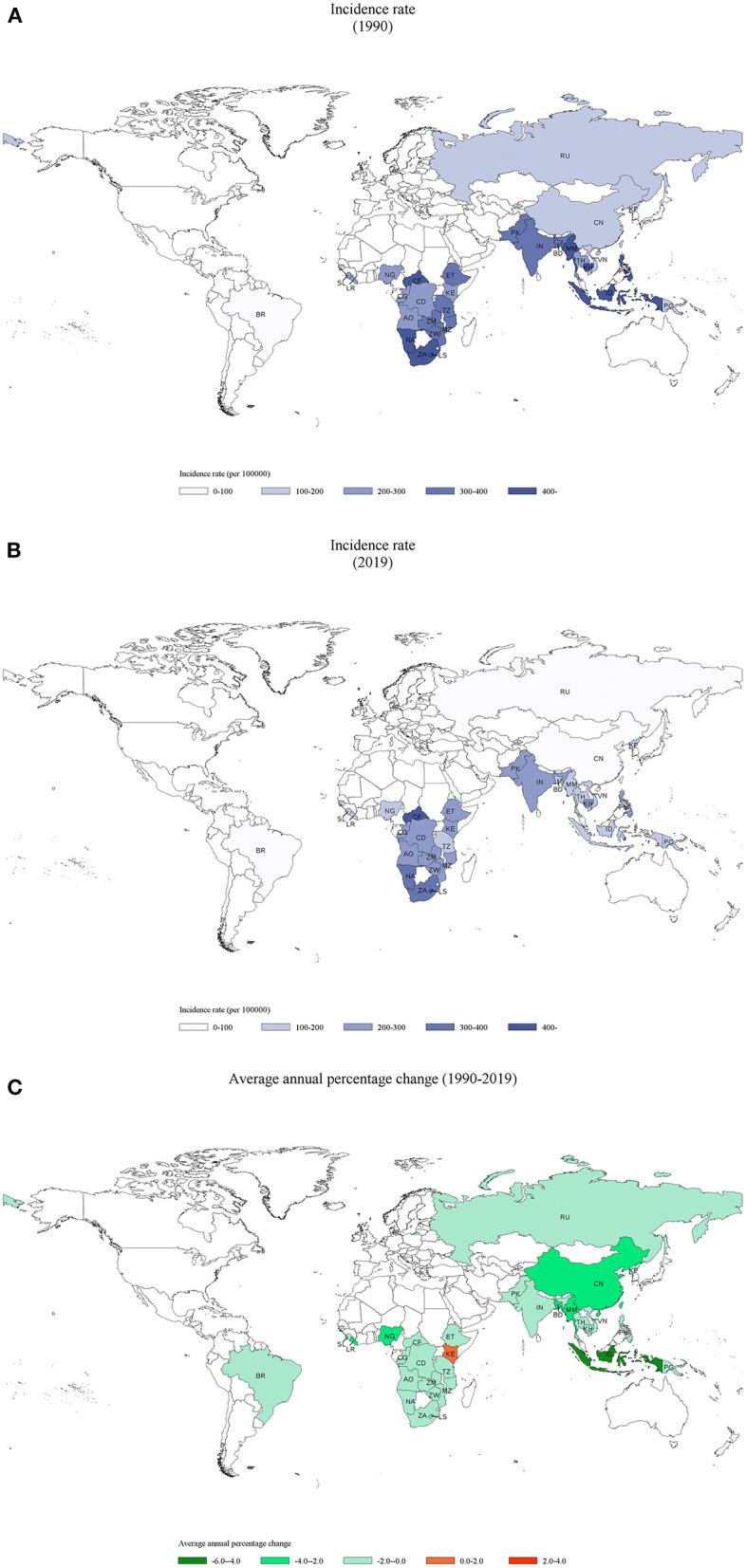
Incidence of tuberculosis in 30 HBCs and its average annual percent change (AAPC): **(A)** the incidence rate in 1990, **(B)** the incidence rate in 2019, **(C)** the AAPC of incidence rate from 1990 to 2019. HBC, high burden country; AO, Angola; BD, Bangladesh; BR, Brazil; KH, Cambodia; CF, Central African Republic; CN, China; CG, Congo; KP, Democratic People's Republic of Korea; CD, Democratic Republic of the Congo; ET, Ethiopia; IN, India; ID, Indonesia; KE, Kenya; LS, Lesotho; LR, Liberia; MZ, Mozambique; MM, Myanmar; NA, Namibia; NG, Nigeria; PK, Pakistan; PG, Papua New Guinea; PH, Philippines; RU, Russian Federation; SL, Sierra Leone; ZA, South Africa; TH, Thailand; TZ, United Republic of Tanzania; VN, Vietnam; ZM, Zambia; ZW, Zimbabwe.

Most of the HBCs are located in areas of Africa and Southeast Asia (Figure 1A). From 1990 to 2019, the incident cases in these HBCs decreased from 7,426,466 to 7,165,583, with the incidence rate decreased from 228.78 (ASR: 368.69) per 100,000 to 148.81 (ASR: 228.74) per 100,000. India had the highest cumulative incidence cases, followed by China, Pakistan, and Indonesia. In 2019, the Central African Republic, Lesotho, Namibia, and South Africa had the highest incidence rates (above 300 per 100,000) compared with that (below 100 per 100,000) in Brazil, China, and Russian Federation (Figure 1B). The incidence in most HBCs decreased with AAPCs ranged between −4.0 (Indonesia) and −0.2% (DR Congo) (all *p* < 0.05) (Supplementary Figure 1). The incidence trends in Lesotho (AAPC: 0%, 95% *CI*: −0.4, 0.3, *p* = 0.8) and South Africa (AAPC: −0.2%, 95% *CI*: −0.5, 0, *p* = 0.1) were stable, while it increased in Kenya (AAPC: 0.1%, 95% *CI*: 0.1, 0.2, *p* < 0.05) (Figure 1C, Table 1, Supplementary Figure 1).

**Table 1:** Incidence, mortality, and MI ratio trends of tuberculosis (TB) in 30 HBCs, 1990–2019.

**Country**	**Incidence**			***P*** **-value**	**Mortality**			***P*** **-value**	**Mortality to incidence ratio**			**Mean SDI**
	**Cumulative cases**	**Mean rate (per 100,000)**	**AAPC (95% CI)**		**Cumulative cases**	**Mean rate (per 100,000)**	**AAPC (95% CI)**		**Mean ratio**	**AAPC (95% CI)**	***P*** **-value**	
Liberia	167,382	173.69	−2.0 (−2.2,−1.9)	<0.05	37,054	38.45	−4.0 (−4.4,−3.6)	<0.05	0.221	−2.3 (−2.6,−1.9)	<0.05	0.20
DR Congo	4,801,325	277.06	−0.2 (−0.3,−0.1)	<0.05	1,405,607	81.11	−2.2 (−2.3,−2.0)	<0.05	0.293	−2 (−2.2,−1.8)	<0.05	0.22
Central African Republic	543,435	468.5	−0.3 (−0.4,−0.3)	<0.05	207,479	178.87	−0.6 (−0.9,−0.4)	<0.05	0.382	−0.3 (−0.5,−0.1)	<0.05	0.23
Sierra Leone	364,566	230.91	−1.1 (−1.2,−1)	<0.05	73,083	46.29	−3.0 (−3.3,−2.8)	<0.05	0.200	−1.9 (−2,−1.7)	<0.05	0.26
Lesotho	249,288	434.03	0 (−0.4, 0.3)	0.8	70,310	122.41	1.3 (1.1, 1.4)	<0.05	0.282	1.3 (0.9, 1.7)	<0.05	0.26
DPR Korea	100,1914	140.10	−0.8 (−0.9,−0.8)	<0.05	102,842	14.38	0.1 (−0.3, 0.4)	0.6	0.103	0.8 (0.6, 0.9)	<0.05	0.28
Thailand	347,3153	180.98	−1.9 (−2.1,−1.7)	<0.05	308,741	16.09	−3.0 (−3.4,−2.6)	<0.05	0.089	−1.2 (−1.6,−0.8)	<0.05	0.33
Angola	139,1932	260.43	−1.2 (−1.3,−1.1)	<0.05	426,534	79.80	−4.4 (−4.7,−4.1)	<0.05	0.306	−3.1 (−3.5,−2.8)	<0.05	0.33
Papua New Guinea	245,689	128.32	−1.4 (−1.5,−1.4)	<0.05	39,264	20.51	−2.4 (−2.5,−2.2)	<0.05	0.160	−0.9 (−1.1,−0.7)	<0.05	0.34
Pakistan	14,670,809	311.32	−1.7 (−2.0,−1.5)	<0.05	2,202,786	46.74	−2.7 (−2.9,−2.5)	<0.05	0.150	−0.9 (−1.1,−0.7)	<0.05	0.34
Cambodia	1,467,757	373.21	−1.4 (−1.5,−1.2)	<0.05	245,529	62.43	−2.9 (−3.0,−2.8)	<0.05	0.167	−1.6 (−1.7,−1.5)	<0.05	0.36
Bangladesh	8,745,421	220.76	−3.3 (−3.3,−3.2)	<0.05	1,374,480	34.70	−4.4 (−4.9,−4.0)	<0.05	0.157	−1.3 (−1.8,−0.7)	<0.05	0.37
Zambia	900,036	259.89	−1.1 (−1.4,−0.7)	<0.05	263,367	76.05	−3.2 (−3.3,−3.1)	<0.05	0.293	−2 (−2.3,−1.7)	<0.05	0.38
Mozambique	1,576,466	269.54	−1.3 (−1.6,−1.1)	<0.05	557,685	95.35	−2.3 (−2.5,−2.1)	<0.05	0.354	−0.9 (−1.4,−0.4)	<0.05	0.39
Namibia	224,351	395.53	−1.4 (−1.5,−1.2)	<0.05	32,165	56.71	−2 (−2.3,−1.8)	<0.05	0.143	−0.7 (−1,−0.4)	<0.05	0.40
Kenya	2,342,310	225.69	0.1 (0.1, 0.2)	<0.05	441,213	42.51	−0.7 (−0.9,−0.6)	<0.05	0.188	−0.9 (−1.1,−0.7)	<0.05	0.41
Indonesia	14,365,998	217.17	−4.0 (−4.2,−3.7)	<0.05	3,092,307	46.75	−2.8 (−3.0,−2.6)	<0.05	0.215	1.3 (1.1, 1.6)	<0.05	0.42
Ethiopia	5,779,543	259.90	−1.7 (−1.8,−1.6)	<0.05	1,658,501	74.58	−5.8 (−5.9,−5.6)	<0.05	0.287	−4.2 (−4.4,−3.9)	<0.05	0.43
Zimbabwe	1,139,198	313.35	−0.8 (−0.9,−0.7)	<0.05	261,793	72.01	1.6 (1.0, 2.2)	<0.05	0.230	2.2 (1.7, 2.8)	<0.05	0.44
Congo	245,622	228.36	−0.7 (−0.9,−0.5)	<0.05	66,997	62.29	−3.1 (−3.4,−2.9)	<0.05	0.273	−2.5 (−3,−2.1)	<0.05	0.46
Nigeria	8,703,451	207.65	−2.2 (−2.3,−2.1)	<0.05	1,624,413	38.76	−3.9 (−4.3,−3.6)	<0.05	0.187	−1.7 (−2,−1.4)	<0.05	0.49
Vietnam	4,076,636	165.27	−0.2 (−0.3,−0.1)	<0.05	723,466	29.33	−2.8 (−2.9,−2.7)	<0.05	0.177	−2.6 (−2.7,−2.4)	<0.05	0.51
Myanmar	3,958,109	280.13	−2.7 (−2.8,−2.6)	<0.05	819,000	57.96	−4.1 (−4.3,−4.0)	<0.05	0.207	−1.4 (−1.6,−1.2)	<0.05	0.53
Philippines	5,563,169	217.84	−1.9 (−2.3,−1.5)	<0.05	843,017	33.01	−1.8 (−2.0,−1.6)	<0.05	0.152	0 (−0.8, 0.8)	1	0.55
India	88,381,415	268.46	−1.3 (−1.4,−1.2)	<0.05	15,504,636	47.10	−3 (−3.5,−2.5)	<0.05	0.175	−1.6 (−2.4,−0.8)	<0.05	0.56
Brazil	1,952,169	35.92	−1.2 (−1.6,−0.8)	<0.05	213,689	3.93	−3 (−3.1,−2.9)	<0.05	0.109	−1.9 (−2.2,−1.5)	<0.05	0.56
China	29,692,404	75.83	−2.3 (−2.4,−2.2)	<0.05	2,594,912	6.63	−5.8 (−6.0,−5.5)	<0.05	0.087	−3.4 (−4.1,−2.8)	<0.05	0.57
UR Tanzania	2,862,012	250.69	−1.8 (−1.9,−1.6)	<0.05	597,459	52.33	−2.5 (−2.8,−2.3)	<0.05	0.209	−0.8(−1,−0.5)	<0.05	0.61
South Africa	5,132,347	369.43	−0.2 (−0.5, 0)	0.1	760,806	54.76	−1.3 (−1.9,−0.7)	<0.05	0.148	−1.1 (−1.9,−0.3)	<0.05	0.62
Russian Federation	5,667,158	129.06	−1.2 (−1.7,−0.6)	<0.05	527,516	12.01	−0.5 (−1.9, 0.9)	0.5	0.093	0.5 (−2.1, 3.2)	0.7	0.75

*MI ratio, mortality to incidence ratio; HBC, high burden country; DR Congo, Democratic Republic of the Congo; DPR Korea, Democratic People's Republic of Korea; UR Tanzania, United Republic of Tanzania*.

The mortality rate decreased from 44 (ASR: 119.01) per 100,000 to 19.71 (ASR: 57.89) per 100,000 from 1990 to 2019. At the county level, India had the highest cumulative deaths of 15,504,636. In 2019, Central African Republic and Lesotho had the highest mortality rates (above 100 per 100,000) compared with that (below 10 per 100,000) in Brazil, China, and Russian Federation. The mortality in most HBCs decreased with AAPCs ranged between −5.8 (Ethiopia) and −0.6% (Central African Republic) (all *p* < 0.05) (Supplementary Figure 1). The mortality trend in DPR Korea (AAPC: 0.1%, 95% *CI*: −0.3, 0.4, *p* = 0.6) and Russian Federation (AAPC: −0.5%, 95% *CI*: −1.9, 0.9, *P* = 0.5) were stable, while it increased in Lesotho and Zimbabwe with AAPC of 1.3% (95% *CI*: 1.1, 1.4, *p* < 0.05) and 1.6% (95% *CI*: 1.0, 2.2, *p* < 0.05), respectively (Table 1, Supplementary Figures 1, 2C).

The mean MI ratio varied from 0.087 (China) to 0.382 (Central African Republic), and decreased in most HBCs, with AAPCs ranging from −4.2 (Ethiopia) to −0.3% (Central African Republic) (all *p* < 0.05). The trends were stable in Philippines (AAPC: 0%, 95% *CI*: −0.8, 0.8, *p* = 1.0) and Russian Federation (AAPC: 0.5%, 95% *CI*: −2.1, 3.2, *p* = 0.7), while it increased in Zimbabwe, Lesotho, Indonesia, and DPR Korea, with the lowest AAPC of 0.8% (all *p* < 0.05) (Table 1, Supplementary Figure 3C). There was no relationship between the mean SDI values and AAPCs (Spearman's correlation: *r* = −0.16, *p* = 0.3883 for incidence; *r* = −0.06, *p* = 0.7602 for mortality; *r* = −0.002, *p* = 0.9916 for MI ratio).

### Disease Burden Change of TB in Age and Sex Subgroups

The incidence and mortality decreased among the three age groups in the majority HBCs (Supplementary Figures 4, 5). Disease burden increased in the group aged 15–59 years in Lesotho (AAPC for incidence: 0.4%, 95% *CI*: 0.2, 0.7, *p* < 0.05; AAPC for mortality: 1.6%, 95% *CI*: 1.4, 1.8, *p* < 0.05). In South Africa, the incidence increased with AAPC of 0.1% (95% *CI*: 0.1, 0.2, *p* < 0.05) among aged 1–14 years group. The incidence trends were stable in the group aged 15–59 years in the Central African Republic, DR Congo, Kenya, and South Africa (*p* > 0.05). Increasing mortality trends was observed in the group aged 15–59 years in Lesotho (AAPC: 1.6%, 95% *CI*: 1.4, 1.8, *p* < 0.05) and Zimbabwe (AAPC: 1.8%, 95% *CI*: 0.9, 2.7, *p* < 0.05), and in the group aged ≥ 60 years in Zimbabwe (AAPC: 0.4%, 95% *CI*: 0.1, 0.7, *p* < 0.05). Among the groups aged 1–14 and 15–59 years in both sexes, Indonesia decreased most in incidence with the lowest AAPCs among those HBCs.

**Figure 2:**
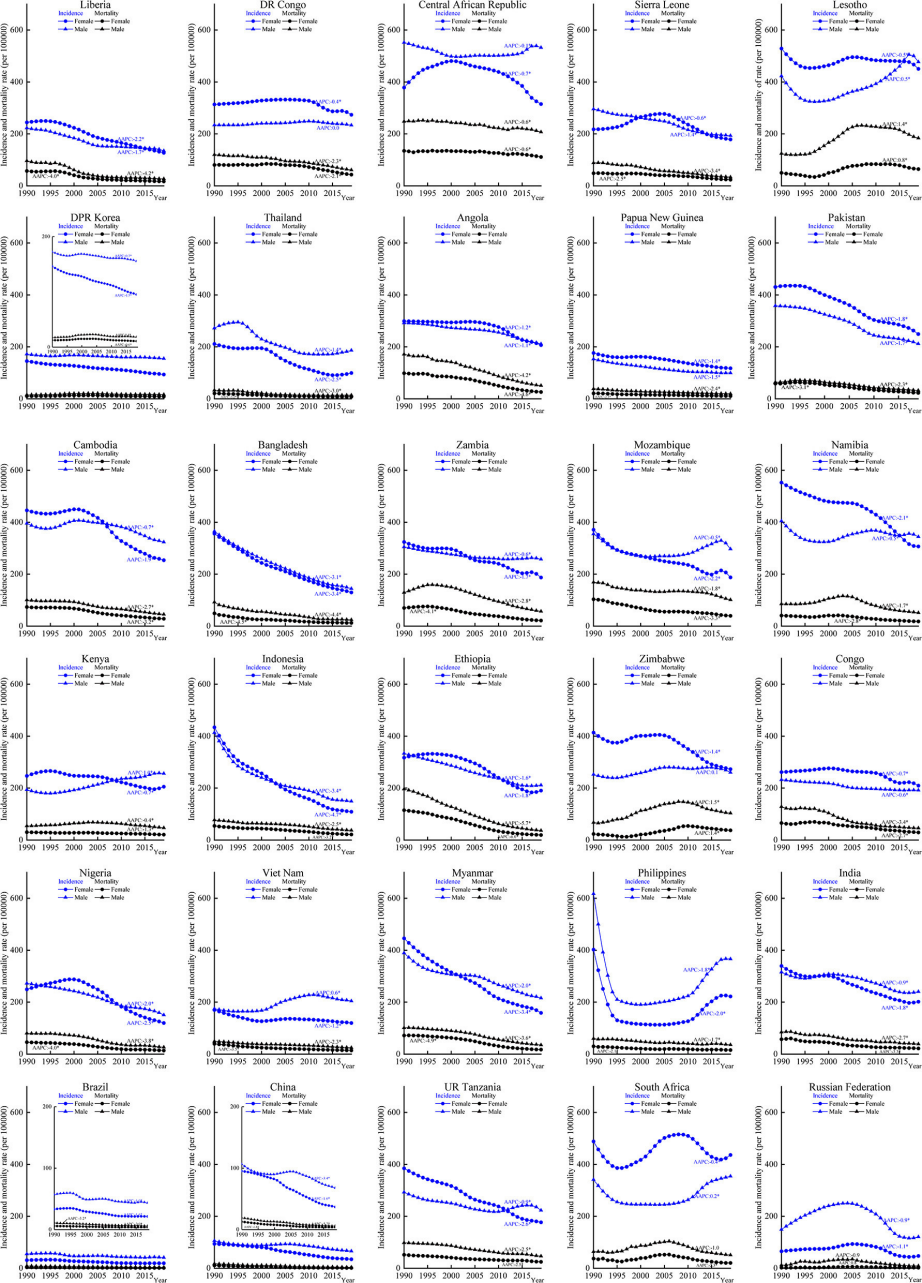
Incidence and mortality of tuberculosis (TB) by different sexes in the 30 HBCs, 1990–2019. HBC, high burden country; AAPC, average annual percent change; *, significant at 0.05 level.

**Figure 3:**
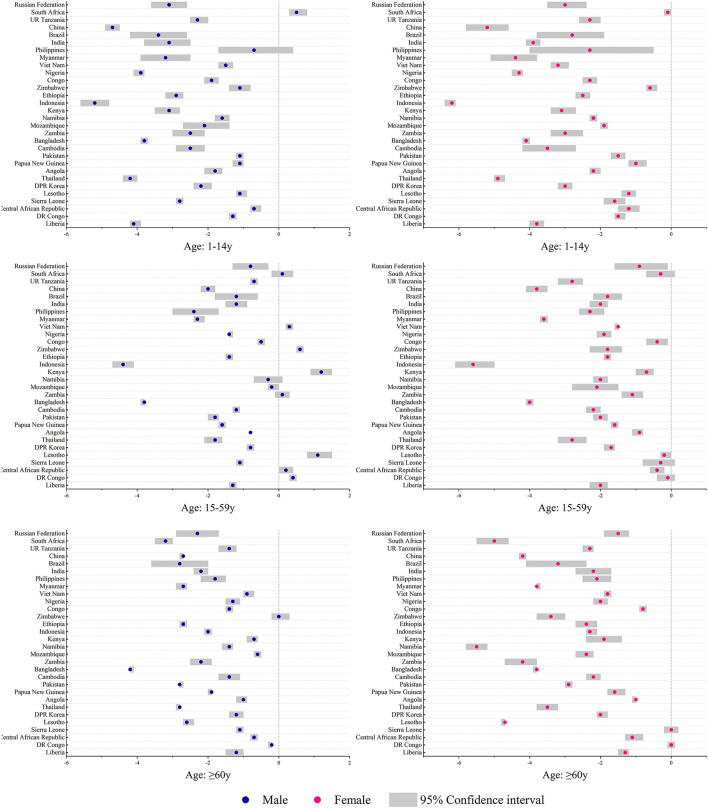
Average annual percent changes of incidence by different age and sex groups in the 30 HBCs, 1990–2019. HBC, high burden country.

There were different incidence trends between men and women in South Africa, Zimbabwe, and Vietnam. The mortality increased in Lesotho (AAPC for men: 1.4%, 95% *CI*: 1.2, 1.5, *p* < 0.05; AAPC for women: 0.8%, 95% *CI*: 0.4, 1.2, *p* < 0.05) and Zimbabwe (AAPC for men: 1.4%, 95% *CI*: 0.8, 2.1, *p* < 0.05; AAPC for women: 1.5%, 95% *CI*: 1.0, 2.0, *p* < 0.05). Women had higher incidence rates in countries, such as DR Congo, Lesotho, Angola, Papua New Guinea, Pakistan, Namibia, Zimbabwe, Congo, UR Tanzania, and South Africa. Most of the above countries are located in Africa (Figure 2). Increasing trends in incidence were observed among men in groups aged 1–14 years (South Africa) and 15–59 years (DR Congo, Lesotho, Kenya, Zimbabwe, and Vietnam) (Figure 3). Mortality rates increased among men (Lesotho and Zimbabwe) and women (Lesotho, Zimbabwe, and Russian Federation) in the group aged 15–59 years, and among both sexes in the group aged ≥60 years (Zimbabwe) (Supplementary Figure 6).

**Figure 4:**
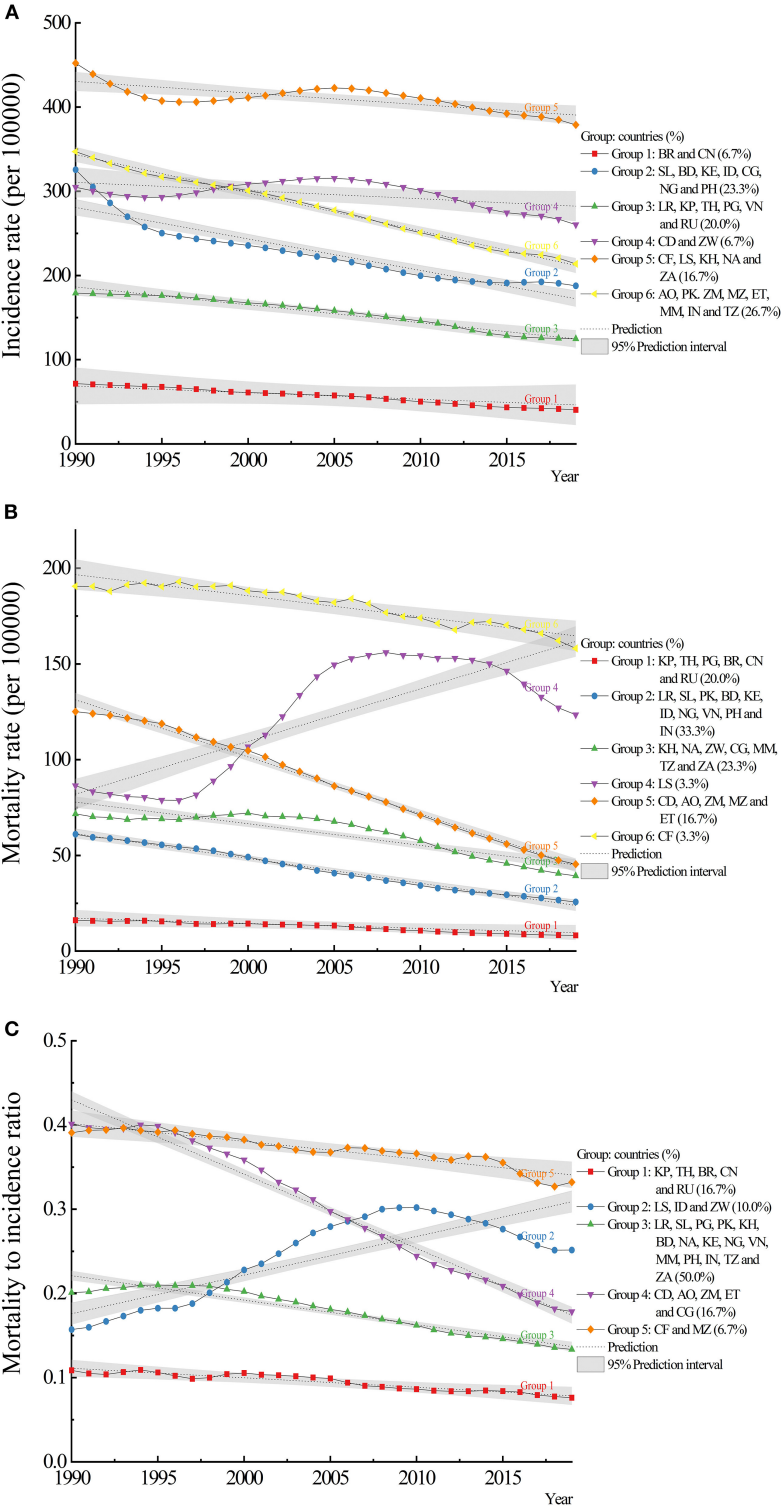
Trajectory analysis of incidence, mortality, and mortality to incidence (MI) ratio of tuberculosis in the 30 HBCs: **(A)** incidence, **(B)** mortality, **(C)** MI ratio. HBC, high burden country; AO, Angola; BD, Bangladesh; BR, Brazil; KH, Cambodia; CF, Central African Republic; CN, China; CG, Congo; KP, Democratic People's Republic of Korea; CD, Democratic Republic of the Congo; ET, Ethiopia; IN, India; ID, Indonesia; KE, Kenya; LS, Lesotho; LR, Liberia; MZ, Mozambique; MM, Myanmar; NA, Namibia; NG, Nigeria; PK, Pakistan; PG, Papua New Guinea; PH, Philippines; RU, Russian Federation; SL, Sierra Leone; ZA, South Africa; TH, Thailand; TZ, United Republic of Tanzania; VN, Vietnam; ZM, Zambia; ZW, Zimbabwe.

The HBCs could be classified as 6 discrete groups by the indicator of incidence (Figure 4A). Group 2, 3, and 6 accounted for 21 (70%) countries. Group 1 (Brazil and China) had the lowest incidence rates while group 5 (Central African Republic, Lesotho, Cambodia, Namibia, and South Africa) had the highest rates. Group 6 had a higher slope value of −4.54 (*p* < 0.0001) while group 1 had the lowest (slope: −0.82, *p* = 0.1293). For mortality, there were 6 groups and one-third of the countries were in group 2 (Figure 4B). Group 1 (DPR Korea, Thailand, Papua New Guinea, Brazil, China, and Russian Federation) had the lowest mortality rates and group 6 (Central African Republic) had the highest rate. Five of the six groups showed decreasing trends of mortality except for group 4 (Lesotho, slope: 2.75, *p* < 0.0001). For MI ratio, group 2 (Lesotho, Indonesia, and Zimbabwe) showed an increasing trend (slope: 0.005, *p* < 0.0001), while other groups showed decreasing trends with slopes ranging from −0.009 to −0.001 (all *p* < 0.0002) (Figure 4C, Supplementary Table 1). Among those HBCs, Brazil and China had relatively lower values of incidence, mortality, and MI ratio. Lower mortality rates contributed to lower MI ratios, which were observed in DPR Korea, Thailand, and Russian Federation.

### Predictions on the Epidemics of TB

Predictions based on the AAPCs, compared with the level in 2015, showed that the change rates of incidence varied from −26.47 to 4.02% in 2025, from −40.02 to 4.54% in 2030, and from −51.09 to 5.06% in 2035. The change rates of mortality varied from −43.30 to −3.36% in 2025, from −57.94 to 4.62% in 2030, and from −68.81 to 13.27% in 2035. The above results revealed that only Brazil and China could reach the milestones set for 2025 with incidence rates below 50 per 100,000 (Table 2).

**Table 2:** Predicted incidence and mortality of TB in 2025, 2030, and 2035 in 30 HBCs.

**Country**	**2015**	**Predicted incidence (95% CI) (per 100,000) and changed (%) by AAPCs**			**Predicted incidence (95% CI) (per 100,000) and changed (%) by trajectory analysis**		
		**2025**	**2030**	**2035**	**2025**	**2030**	**2035**
Liberia	144.41	116.15 (114.73, 116.86)	104.99 (102.65, 106.17)	94.9 (91.85, 96.46)	118.35 (114.67, 122.03)	107.71 (100.96, 114.46)	97.08 (87.26, 106.89)
		−19.57	−27.3	−34.28	−18.05	−25.41	−32.77
DR Congo	263.47	250.88 (249.38, 252.4)	248.39 (245.66, 251.14)	245.91 (242, 249.88)	248.05 (241.66, 254.45)	243.17 (231.44, 254.9)	238.28 (221.22, 255.34)
		−4.78	−5.72	−6.66	−5.85	−7.7	−9.56
Central African Republic	447.17	414.02 (411.54, 414.02)	407.85 (403.37, 407.85)	401.77 (395.37, 401.77)	413.31 (409.28, 417.33)	406.43 (399.05, 413.81)	399.55 (388.82, 410.29)
		−7.41	−8.79	−10.15	−7.57	−9.11	−10.65
Sierra Leone	195.46	174.13 (173.08, 175.19)	164.76 (162.94, 166.6)	155.9 (153.39, 158.44)	163.65 (160.27, 167.04)	144.96 (138.76, 151.17)	126.27 (117.25, 135.3)
		−10.91	−15.71	−20.24	−16.27	−25.84	−35.4
Lesotho	470.11	463.81 (452.79, 472.22)	463.81 (443.81, 479.35)	463.81 (435, 486.58)	455.56 (451.53, 459.59)	448.69 (441.3, 456.07)	441.81 (431.07, 452.55)
		−1.34	−1.34	−1.34	−3.1	−4.56	−6.02
DPR Korea	130.05	118.34 (117.63, 118.34)	113.68 (112.43, 113.68)	109.21 (107.46, 109.21)	111.42 (107.74, 115.1)	100.78 (94.03, 107.53)	90.15 (80.33, 99.96)
		−9	−12.59	−16.02	−14.33	−22.51	−30.68
Thailand	130.97	126.6 (125.06, 128.16)	115.02 (112.47, 117.63)	104.5 (101.15, 107.97)	129.28 (125.6, 132.96)	118.65 (111.9, 125.4)	108.01 (98.19, 117.83)
		−3.34	−12.18	−20.21	−1.29	−9.41	−17.53
Angola	227.56	194.38 (193.21, 195.57)	183 (180.97, 185.05)	172.28 (169.51, 175.09)	181.71 (178.53, 184.89)	158.98 (153.14, 164.81)	136.25 (127.76, 144.73)
		−14.58	−19.58	−24.29	−20.15	−30.14	−40.13
Papua New Guinea	112.92	99.6 (99, 99.6)	92.82 (91.79, 92.82)	86.5 (85.11, 86.5)	95.63 (91.95, 99.31)	84.99 (78.24, 91.74)	74.36 (64.54, 84.17)
		−11.8	−17.8	−23.4	−15.31	−24.73	−34.15
Pakistan	257.87	207.65 (203.88, 210.2)	190.59 (184.29, 194.9)	174.93 (166.58, 180.72)	202.88 (199.69, 206.06)	180.14 (174.31, 185.98)	157.41 (148.93, 165.9)
		−19.47	−26.09	−32.16	−21.32	−30.14	−38.96
Cambodia	312.52	264.8 (263.19, 268.04)	246.77 (244.03, 252.34)	229.98 (226.27, 237.55)	279.92 (275.89, 283.95)	273.05 (265.66, 280.43)	266.17 (255.43, 276.91)
		−15.27	−21.04	−26.41	−10.43	−12.63	−14.83
Bangladesh	152.64	112.23 (112.23, 112.93)	94.9 (94.9, 95.98)	80.24 (80.24, 81.58)	114.84 (111.45, 118.23)	96.15 (89.94, 102.36)	77.46 (68.43, 86.49)
		−26.47	−37.83	−47.43	−24.76	−37.01	−49.25
Zambia	231.41	207.96 (204.2, 213.06)	196.77 (190.3, 205.7)	186.18 (177.35, 198.6)	194.95 (191.77, 198.13)	172.22 (166.39, 178.05)	149.49 (141, 157.98)
		−10.13	−14.97	−19.55	−15.76	−25.58	−35.4
Mozambique	255.26	221.96 (217.94, 224.67)	207.9 (201.06, 212.59)	194.74 (185.48, 201.15)	212.81 (209.63, 216)	190.08 (184.25, 195.92)	167.35 (158.86, 175.84)
		−13.05	−18.55	−23.71	−16.63	−25.53	−34.44
Namibia	348.57	298.53 (296.72, 302.18)	278.21 (275.12, 284.48)	259.27 (255.1, 267.82)	316.63 (312.61, 320.66)	309.76 (302.38, 317.14)	302.88 (292.14, 313.62)
		−14.36	−20.19	−25.62	−9.16	−11.13	−13.11
Kenya	223.4	232.38 (232.38, 233.77)	233.54 (233.54, 236.12)	234.71 (234.71, 238.49)	208.56 (205.18, 211.95)	189.87 (183.67, 196.08)	171.18 (162.15, 180.21)
		4.02	4.54	5.06	−6.64	−15.01	−23.38
Indonesia	137.72	101.32 (100.06, 103.23)	82.61 (80.74, 85.5)	67.36 (65.15, 70.81)	107.01 (103.62, 110.4)	88.32 (82.11, 94.53)	69.63 (60.6, 78.66)
		−26.43	−40.02	−51.09	−22.3	−35.87	−49.44
Ethiopia	202.86	181.43 (180.33, 182.54)	166.53 (164.67, 168.4)	152.85 (150.38, 155.35)	173.82 (170.63, 177)	151.09 (145.25, 156.92)	128.35 (119.87, 136.84)
		−10.56	−17.91	−24.65	−14.32	−25.52	−36.73
Zimbabwe	285.85	254.27 (252.74, 255.81)	244.26 (241.57, 246.98)	234.65 (230.89, 238.46)	260.96 (254.56, 267.36)	256.08 (244.35, 267.81)	251.19 (234.13, 268.25)
		−11.05	−14.55	−17.91	−8.71	−10.41	−12.13
Congo	206.14	192.45 (190.14, 194.79)	185.81 (181.73, 189.97)	179.4 (173.7, 185.26)	178.31 (174.92, 181.69)	159.62 (153.41, 165.83)	140.93 (131.9, 149.96)
		−6.64	−9.86	−12.97	−13.5	−22.57	−31.63
Nigeria	157.33	117.94 (117.22, 118.66)	105.52 (104.34, 106.72)	94.42 (92.88, 95.97)	112.35 (108.97, 115.74)	93.67 (87.46, 99.87)	74.98 (65.95, 84.01)
		−25.04	−32.93	−39.99	−28.59	−40.46	−52.34
Vietnam	170.88	160.15 (159.19, 161.12)	158.56 (156.82, 160.32)	156.98 (154.48, 159.52)	149.33 (145.64, 153.01)	138.69 (131.94, 145.44)	128.05 (118.23, 137.87)
		−6.28	−7.21	−8.13	−12.61	−18.84	−25.06
Myanmar	208.91	158.01 (157.03, 158.98)	137.8 (136.25, 139.36)	120.17 (118.21, 122.16)	158.93 (155.75, 162.11)	136.2 (130.36, 142.03)	113.47 (104.98, 121.96)
		−24.36	−34.04	−42.48	−23.92	−34.8	−45.68
Philippines	265.91	263.1 (256.73, 269.6)	239.04 (228.53, 249.98)	217.18 (203.43, 231.79)	272.77 (269.38, 276.15)	254.08 (247.87, 260.29)	235.39 (226.36, 244.42)
		−1.06	−10.1	−18.33	2.58	−4.45	−11.48
India	223.37	205.04 (203.79, 206.29)	192.05 (189.92, 194.2)	179.89 (176.99, 182.83)	194.5 (191.32, 197.69)	171.77 (165.94, 177.61)	149.04 (140.56, 157.53)
		−8.21	−14.02	−19.47	−12.92	−23.1	−33.28
Brazil**	30.21	28.04 (27.36, 28.72)	26.39 (25.24, 27.59)	24.85 (23.29, 26.51)	25.21 (18.84, 31.58)	21.1 (9.42, 32.77)	16.99 (0, 33.97)
		−7.18*	−12.64	−17.74	−16.55*	−30.16	−43.76
China**	56.54	44.26 (43.99, 44.54)	39.4 (38.96, 39.85)	35.07 (34.5, 35.65)	45.96 (39.59, 52.33)	41.85 (30.17, 53.53)	37.74 (20.76, 54.73)
		−21.72*	−30.31	−37.97	−18.71*	−25.98	−33.25
UR Tanzania	215.37	178.79 (177.7, 180.99)	163.27 (161.45, 166.97)	149.1 (146.68, 154.03)	172.1 (168.92, 175.28)	149.37 (143.54, 155.21)	126.64 (118.15, 135.13)
		−16.98	−24.19	−30.77	−20.09	−30.64	−41.2
South Africa	383.27	391.76 (384.75, 396.49)	387.86 (375.22, 396.49)	384 (365.94, 396.49)	388.24 (384.22, 392.27)	381.37 (373.99, 388.75)	374.49 (363.76, 385.23)
		2.22	1.2	0.19	1.3	−0.5	−2.29
Russian Federation	82.55	75.91 (73.64, 78.72)	71.47 (67.59, 76.39)	67.28 (62.03, 74.12)	68.85 (65.17, 72.53)	58.21 (51.46, 64.96)	47.58 (37.76, 57.4)
		−8.04	−13.42	−18.5	−16.6	−29.49	−42.36
Liberia**	25.06	17.46 (17.03, 17.9)	14.23 (13.59, 14.9)	11.61 (10.86, 12.4)	14.53 (13.63, 15.43)	8.05 (6.41, 9.7)	1.58 (0, 3.97)
		−30.33	−43.22	−53.67	−42.02	−67.88	−93.7
DR Congo	64.65	45.65 (45.37, 46.21)	40.84 (40.39, 41.77)	36.54 (35.95, 37.76)	34.29 (33.02, 35.55)	19.39 (17.06, 21.71)	4.49 (1.1, 7.87)
		−29.39	−36.83	−43.48	−46.96	−70.01	−93.05
Central African Republic	170.34	152.54 (149.8, 154.39)	148.02 (143.18, 151.33)	143.63 (136.85, 148.33)	151.52 (148.68, 154.36)	146 (140.79, 151.2)	140.48 (132.91, 148.05)
		−10.45	−13.1	−15.68	−11.05	−14.29	−17.53
Sierra Leone	33.51	23.61 (23.18, 23.91)	20.28 (19.6, 20.74)	17.41 (16.57, 18)	20.58 (19.68, 21.48)	14.1 (12.46, 15.75)	7.63 (5.23, 10.02)
		−29.54	−39.48	−48.05	−38.59	−57.92	−77.23
Lesotho	146.4	133.5 (131.92, 134.29)	142.4 (139.34, 143.96)	151.9 (147.17, 154.32)	140.04 (137.2, 142.88)	153.8 (148.59, 159)	167.55 (159.98, 175.12)
		−8.81	−2.73	3.76	−4.34	5.05	14.45
DPR Korea	13.38	12.53 (12.23, 12.76)	12.59 (12.05, 13.02)	12.66 (11.87, 13.28)	10.66 (9.5, 11.82)	9.16 (7.04, 11.29)	7.66 (4.57, 10.76)
		−6.35	−5.9	−5.38	−20.33	−31.54	−42.75
Thailand	10.91	9.31 (9.08, 9.54)	7.99 (7.64, 8.36)	6.86 (6.42, 7.33)	9.38 (8.22, 10.54)	7.88 (5.75, 10)	6.38 (3.29, 9.47)
		−14.67	−26.76	−37.12	−14.02	−27.77	−41.52
Angola**	48.11	29.26 (28.71, 29.81)	23.36 (22.57, 24.18)	18.66 (17.74, 19.61)	20.45 (19.18, 21.72)	5.55 (3.22, 7.87)	0 (0, 0)
		−39.18	−51.44	−61.21	−57.49	−88.46	−100*
Papua New Guinea	16.97	13.25 (13.17, 13.42)	11.74 (11.61, 12.01)	10.4 (10.23, 10.74)	13.54 (12.38, 14.7)	12.04 (9.91, 14.16)	10.54 (7.45, 13.63)
		−21.92	−30.82	−38.72	−20.21	−29.05	−37.89
Pakistan	32.75	23.49 (23.21, 23.78)	20.49 (20.03, 20.96)	17.87 (17.29, 18.46)	19.92 (19.02, 20.81)	13.44 (11.79, 15.09)	6.96 (4.57, 9.36)
		−28.27	−37.44	−45.44	−39.18	−58.96	−78.75
Cambodia	43.32	30.82 (30.63, 31.01)	26.6 (26.3, 26.91)	22.96 (22.59, 23.35)	29.99 (28.91, 31.06)	24.33 (22.36, 26.3)	18.68 (15.81, 21.54)
		−28.86	−38.6	−47	−30.77	−43.84	−56.88
Bangladesh**	20.04	14.05 (13.62, 14.41)	11.22 (10.59, 11.75)	8.96 (8.24, 9.58)	10.64 (9.74, 11.53)	4.16 (2.51, 5.81)	0 (0, 0.08)
		−29.89	−44.01	−55.29	−46.91	−79.24	−100*
Zambia**	48.57	32.31 (32.11, 32.51)	27.46 (27.15, 27.77)	23.34 (22.95, 23.73)	21.39 (20.12, 22.66)	6.49 (4.16, 8.82)	0 (0, 0)
		−33.48	−43.46	−51.95	−55.96	−86.64	−100*
Mozambique	84.15	60.35 (59.61, 61.09)	53.72 (52.52, 54.94)	47.82 (46.28, 49.41)	51.51 (50.24, 52.78)	36.61 (34.28, 38.94)	21.71 (18.32, 25.1)
		−28.28	−36.16	−43.17	−38.79	−56.49	−74.2
Namibia	39.72	30.48 (29.92, 30.85)	27.55 (26.63, 28.17)	24.9 (23.71, 25.73)	27.62 (26.54, 28.69)	21.96 (19.99, 23.93)	16.3 (13.44, 19.17)
		−23.26	−30.64	−37.31	−30.46	−44.71	−58.96
Kenya	39.08	32.55 (32.16, 32.74)	31.42 (30.73, 31.77)	30.34 (29.38, 30.83)	26.18 (25.28, 27.07)	19.7 (18.05, 21.35)	13.23 (10.83, 15.62)
		−16.71	−19.6	−22.36	−33.01	−49.59	−66.15
Indonesia	33.36	24.99 (24.69, 25.31)	21.69 (21.2, 22.18)	18.82 (18.21, 19.44)	21.87 (20.97, 22.77)	15.39 (13.75, 17.04)	8.92 (6.52, 11.31)
		−25.09	−34.98	−43.59	−34.44	−53.87	−73.26
Ethiopia**	34.5	19.56 (19.44, 19.81)	14.51 (14.34, 14.85)	10.76 (10.58, 11.13)	10.12 (8.85, 11.39)	0 (0, 0)	0 (0, 0)
		−43.3	−57.94	−68.81	−70.67	−100*	−100*
Zimbabwe	78.83	76.18 (73.52, 78.92)	82.47 (77.27, 87.99)	89.29 (81.21, 98.11)	62.47 (61.4, 63.55)	56.82 (54.85, 58.78)	51.16 (48.3, 54.02)
		−3.36	4.62	13.27	−20.75	−27.92	−35.1
Congo	42.67	31.24 (30.67, 31.63)	26.69 (25.79, 27.3)	22.8 (21.7, 23.57)	30.95 (29.88, 32.02)	25.29 (23.33, 27.26)	19.64 (16.78, 22.5)
		−26.79	−37.45	−46.57	−27.47	−40.73	−53.97
Nigeria**	25.11	15.87 (15.47, 16.17)	13 (12.42, 13.46)	10.66 (9.97, 11.2)	12.37 (11.47, 13.27)	5.9 (4.25, 7.54)	0 (0, 1.81)
		−36.8	−48.23	−57.55	−50.74	−76.5	−100*
Vietnam**	22.49	16.53 (16.43, 16.64)	14.35 (14.18, 14.51)	12.45 (12.24, 12.65)	11.84 (10.94, 12.73)	5.36 (3.71, 7.01)	0 (0, 1.28)
		−26.5	−36.19	−44.64	−47.35	−76.17	−100*
Myanmar	31.43	20.26 (20.01, 20.39)	16.44 (16.06, 16.63)	13.33 (12.89, 13.56)	19.26 (18.19, 20.33)	13.61 (11.64, 15.57)	7.95 (5.09, 10.81)
		−35.54	−47.69	−57.59	−38.72	−56.7	−74.71
Philippines	28.91	23.58 (23.3, 23.87)	21.54 (21.06, 22.02)	19.67 (19.04, 20.32)	18.53 (17.63, 19.43)	12.05 (10.41, 13.7)	5.58 (3.18, 7.97)
		−18.44	−25.49	−31.96	−35.9	−58.32	−80.7
India	33.88	25.55 (24.77, 26.35)	21.94 (20.73, 23.22)	18.84 (17.35, 20.46)	22.9 (22, 23.8)	16.43 (14.78, 18.07)	9.95 (7.56, 12.35)
		−24.59	−35.24	−44.39	−32.41	−51.51	−70.63
Brazil**	2.77	2.11 (2.1, 2.13)	1.81 (1.79, 1.83)	1.56 (1.53, 1.58)	0.74 (0, 1.9)	0 (0, 1.37)	0 (0, 0.83)
		−23.83	−34.66	−43.68	−73.29	−100*	−100*
China**	3.01	1.8 (1.78, 1.84)	1.34 (1.31, 1.39)	0.99 (0.96, 1.04)	0.78 (0, 1.94)	0 (0, 1.41)	0 (0, 0.88)
		−40.2	−55.48	−67.11	−74.09	−100*	−100*
UR Tanzania	42.31	30.41 (29.85, 30.79)	26.8 (25.9, 27.41)	23.61 (22.47, 24.4)	28.61 (27.54, 29.69)	22.96 (20.99, 24.92)	17.3 (14.44, 20.16)
		−28.13	−36.66	−44.2	−32.38	−45.73	−59.11
South Africa	42.31	32.89 (31.71, 34.1)	30.8 (28.81, 32.93)	28.85 (26.17, 31.79)	28.79 (27.71, 29.86)	23.13 (21.16, 25.1)	17.47 (14.61, 20.34)
		−22.26	−27.2	−31.81	−31.95	−45.33	−58.71
Russian Federation	7.34	5.33 (4.89, 5.79)	5.19 (4.44, 6.06)	5.06 (4.04, 6.33)	3.69 (2.53, 4.85)	2.19 (0.07, 4.32)	0.69 (0, 3.79)
		−27.38	−29.29	−31.06	−49.73	−70.16	−90.6

*HBC, high burden country; DR Congo, Democratic Republic of the Congo; DPR Korea, Democratic People's Republic of Korea; UR Tanzania, United Republic of Tanzania*.

* *, Goal achieved regarding the targets set for TB by the WHO*.

** *, Countries that may achieve the targets by different predictions*.

Predictions based on trajectory analysis showed that the change rates of incidence varied from −28.59 to 2.58% in 2025, from −40.46 to −0.50% in 2030, and from −52.34 to −2.29% in 2035. The change rates of mortality varied from −74.09 to −4.34% in 2025, from −100 to 5.05% in 2030, and from −100 to 14.45% in 2035. Reduction targets set for deaths could be achieved by Brazil, China, and Ethiopia in 2030, and Angola, Bangladesh, Brazil, China, Ethiopia, Nigeria, Vietnam, and Zambia in 2035 (Table 2).

## Discussion

This study provided alternative evidence on the TB epidemic in the 30 HBCs besides the WHO. From 1990 to 2019, the disease burden of TB decreased in most HBCs (AAPCs of incidence ranged from −4.0 to −0.2%, AAPCs of mortality ranged from −5.8 to −0.6%, AAPCs of MI ratios ranged from −4.2 to −0.3%). The trajectory models also indicated these. In addition, the WHO estimated that the average decline rate of global incidence was 1.7% per year (2000–2019), and 45% of mortality. Compared with this study, specifically, the WHO reported that the incidence trend (2000–2019) in Liberia increased, while it decreased in Kenya, and was stable in Bangladesh, Central African Republic, DPR Korea, DR Congo, Nigeria, Pakistan, and Papua New Guinea. The mortality was decreased in the majority HBCs, except in countries, such as Central African Republic, Lesotho, Namibia, and Zambia (stable) and Congo, Liberia, and Namibia (increasing) (2).

The WHO reported that the highest burden of TB was among adult men (accounting for 56% of all cases in 2019) (2). Results from this study were similar except for countries, such as DR Congo, Angola, Papua New Guinea, Pakistan, Zimbabwe, Congo, and South Africa, among which higher incidence rates were observed among women in the same year. Globally, 53% of the HIV-negative adults who died from TB were men, 31% were women in 2019 (2). Moreover, men had higher mortality rates in this study. Additionally, the higher incidence and mortality rates occurred in adult groups and increase with age (Supplementary Figures 4, 5), which confirms that TB mostly affects adults in their most productive years (19).

To parallel the global targets, incidence and mortality rates at the country level instead of age-standardized ones were chosen in addressing the purpose of this study. Both predictions showed that the reduction targets of incidence and mortality set by the WHO were difficult to be achieved. Consistent with the above results, countries in most of the WHO regions were not on track to reach the 2020 milestones (2), and the global annual decline in the incidence needs to accelerate to 4–5% per year by 2020 and 10% by 2025, and even an average of 17% set for 2030 (2). More importantly, this was estimated at the global level instead of the country level. Remarkably, more than half of the global TB cases and two-thirds of the multidrug-resistant TB (MDR-TB) cases were borne by Brazil, Russian Federation, India, China, and South Africa (20). For those HCBs, there were more challenges and longer ways toward meeting these ambitious targets (21–24).

The WHO has underscored the significance of monitoring the status of the TB epidemic and progress in financing and implementation of the response at global, regional, and country levels (19). The up-to-date information is essential to track the progress of control efforts (4). The programmatic management of latent tuberculosis infection (LTBI) is one of the crucial interventions to decrease the incidence (25). In addition, addressing the MDR-TB epidemic is crucial to reducing morbidity, mortality, and healthcare related costs (26, 27). The long-term socio-economic effects will further drive risk factors associated with TB, such as poverty, malnutrition, and poor living conditions (24). Unexpected factors would affect the elimination of TB. For example, the pandemic of COVID-19 has resulted in a crisis in public health and challenged healthcare systems worldwide (28–30), which threatens to reverse the gains made over recent years (2). Upon these challenging targets, intensified national and global efforts, such as technical assistance and cooperation, promotion of integrated, patient-centered care and prevention, continued scale-up of early diagnosis, and proper treatment for all forms of TB, bold policies and supportive systems, and global surveillance are the appropriate and responsible way forward (9, 20, 21, 31).

From trajectory analysis, the characteristics of subgroups could be summarized and analyzed, and comparisons could be made between subgroups. For example, treating the active TB or LTBI cases, protecting the susceptible population, and reducing the transmission of the disease are the principle in the subgroup of Central African Republic, Lesotho, Cambodia, Namibia, and South Africa with relatively higher incidence rates. The Central African Republic and Lesotho with a high burden of mortality should pay more attention to reducing deaths, which illustrates large inequities in access to diagnosis and treatment (21). Developed countries achieved remarkable reductions by pursuing universal access to healthcare and social protection while rapidly improving the nutrition and economic conditions in addition to delivering adequate services (9). Although reductions seem also possible with current interventions among HBCs, additional interventions, adapted to country-specific epidemiology, and health systems, are needed (23).

High-level leadership is listed as a key element of a national-level mechanism regarding a multi-sectoral accountability framework for TB (2). Brazil created the Brazilian TB Research Network in 2001 (32) and proposed the Research Agenda in 2015 (33), both of which represented a novel form of collaboration among academia, the government, civil society, and the national industrial sector to the community (34). Additionally, in 2017, the National Plan for Eliminating Tuberculosis for Brazil was launched (35). In China, the incidence rate decreased annually from 109 per 100,000 in 2000 to 64 per 100,000 in 2016 (36). The newest National Action Plan for TB Control for 2019–2022 was proposed (incidence decreases to under 55 per 100,000 and mortality decreases to under 3 per 100,000) (37), as defined in the Healthy China Action Plan (2019–2030) (38).

Thailand has established a universal health coverage scheme and a strong primary healthcare system since 2002 (39). Thailand stands out as having a relatively high service coverage index of 80 among HBCs and a low level of catastrophic health expenditures (2% of households) (2). Brazil, China, Russian Federation, and Thailand had high levels of treatment coverage (>80%) in 2019, while it was 50% or less in the Central African Republic and Nigeria (2). The observed results were questionable in DPR Korea, in which the highest TB burden outside of sub-Saharan Africa was reported (2). Since the famines of the 1990s, the TB burden in DPR Korea had risen dramatically (40, 41). The WHO reported an incidence rate of 513 per 100,000 in 2019, which was only 124.18 per 100,000 in this study. The incidence trend (2000–2019) showed a horizontal line and the morality trend was unavailable in the WHO report (2), which was different from this study. The differences illustrated the importance of data sources and model estimations.

The following major categories were used by the WHO to estimate incidences and mortalities: prevalence surveys, notifications adjusted by a standard factor, inventory/capture-recapture studies and case notification data combined with expert opinions, vital registration systems or mortality surveys, and deriving from multiplying estimates of incidence by estimates of the case fatality rate (42). The GBD study used the data available from annual case notifications, prevalence surveys, population based tuberculin surveys, vital registration data, verbal autopsies, and estimated cause-specific mortality rates among individuals (4, 43). It was found that even the 20 countries with the highest number of incident TB cases in 2016 were different between the two estimations (4). In the newest global tuberculosis report, countries, such as Cambodia, Russian Federation, and Zimbabwe have been replaced by Gabon, Mongolia, and Uganda in the HCBs list for the period of 2021–2025 (3). The disparities were mainly due to the different methodological approaches used and interpretations of data (44). Additionally, the discrepancy calls attention to the modeling approaches and more importantly the high-quality data. It is possible that different independent estimates would contribute to the reliability of the collected data.

This study has several limitations. First, although data from the GBD study are considered widely accepted, the accuracy of data collected, extracted, and reported is still improving. Further cross-sectional and longitudinal studies in those HBCs along with other countries are needed to follow the epidemic trend of TB. Second, predictions in this study have certain assumptions, such as the trends, population size, influencing factors, and interventions were kept the same. Potential factors (e.g., LTBI, MDR-TB, and HIV co-infection) associated with the epidemics were not involved (45). Third, no subdivisions, such as LTBI, MDR-TB, or extensively drug-resistant TB were analyzed.

## Conclusions

Although significant progress has been made on reducing the TB burden in those HBCs during the past three decades, the findings suggest that, if current trends continue, achieving the global targets of end TB at the country level among those HBCs is challenging. Progress needs to be accelerated to achieve the global targets.

## Data Availability Statement

Publicly available datasets were analyzed in this study. This data can be found here: http://ghdx.healthdata.org/gbd-results-tool.

## Author Contributions

CD and KX conceived and designed the study. MH, YS, WG, SW, and XF collected data. CD, MH, YS, WG, SW, XF, and ZZ cleaned, analyzed the data, and interpreted the results. CD and YZ wrote the first draft. CD, YZ, and KX contributed to figures and manuscript preparation. All the authors critically revised the manuscript and accessed the final approval for publication.

## Funding

This study was supported by the National Key Research and Development Program of China (2021YFC2301800); the Medical and Health Science and Technology Project of Zhejiang Province (2022KY743); the National Science and Technology Major Project (2017ZX10105001 and 2018ZX10715014); and the Lishui Science and Technology Bureau (2018ZDHZ10). The GBD was partially funded by the Bill & Melinda Gates Foundation.

## Conflict of Interest

The authors declare that the research was conducted in the absence of any commercial or financial relationships that could be construed as a potential conflict of interest.

## Publisher's Note

All claims expressed in this article are solely those of the authors and do not necessarily represent those of their affiliated organizations, or those of the publisher, the editors and the reviewers. Any product that may be evaluated in this article, or claim that may be made by its manufacturer, is not guaranteed or endorsed by the publisher.
