# Supplementary figures and images for: Epidemic Trends in High Tuberculosis Burden Countries During the Last Three Decades and Feasibility of Achieving the Global Targets at the Country Level
Source: Front Med (Lausanne). 2022 Mar 3;9:798465. doi: 10.3389/fmed.2022.798465 (PMC8927670; doi:10.3389/fmed.2022.798465)

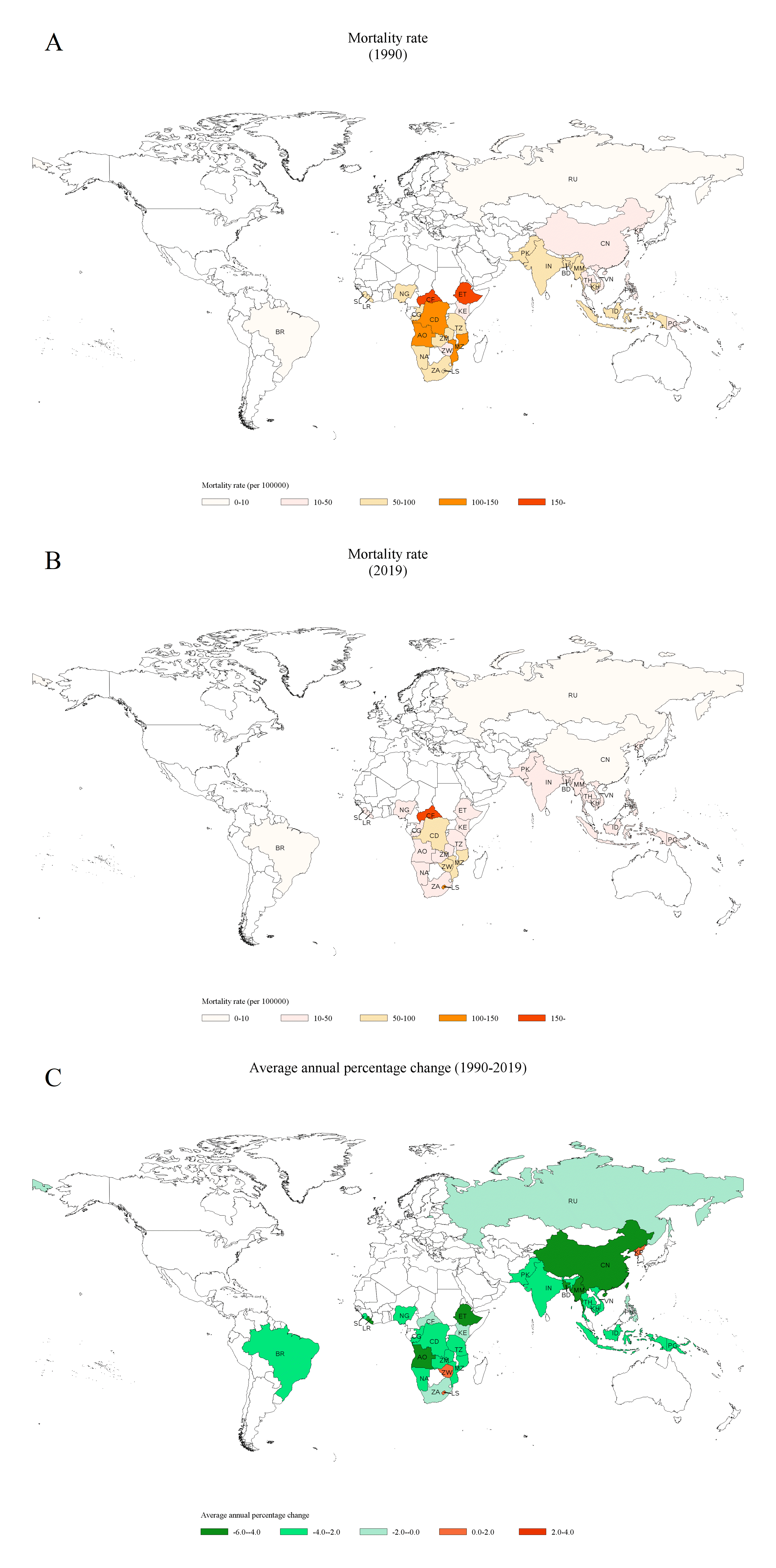

Supplement: Supplementary file 2 [file Image_2.TIF]

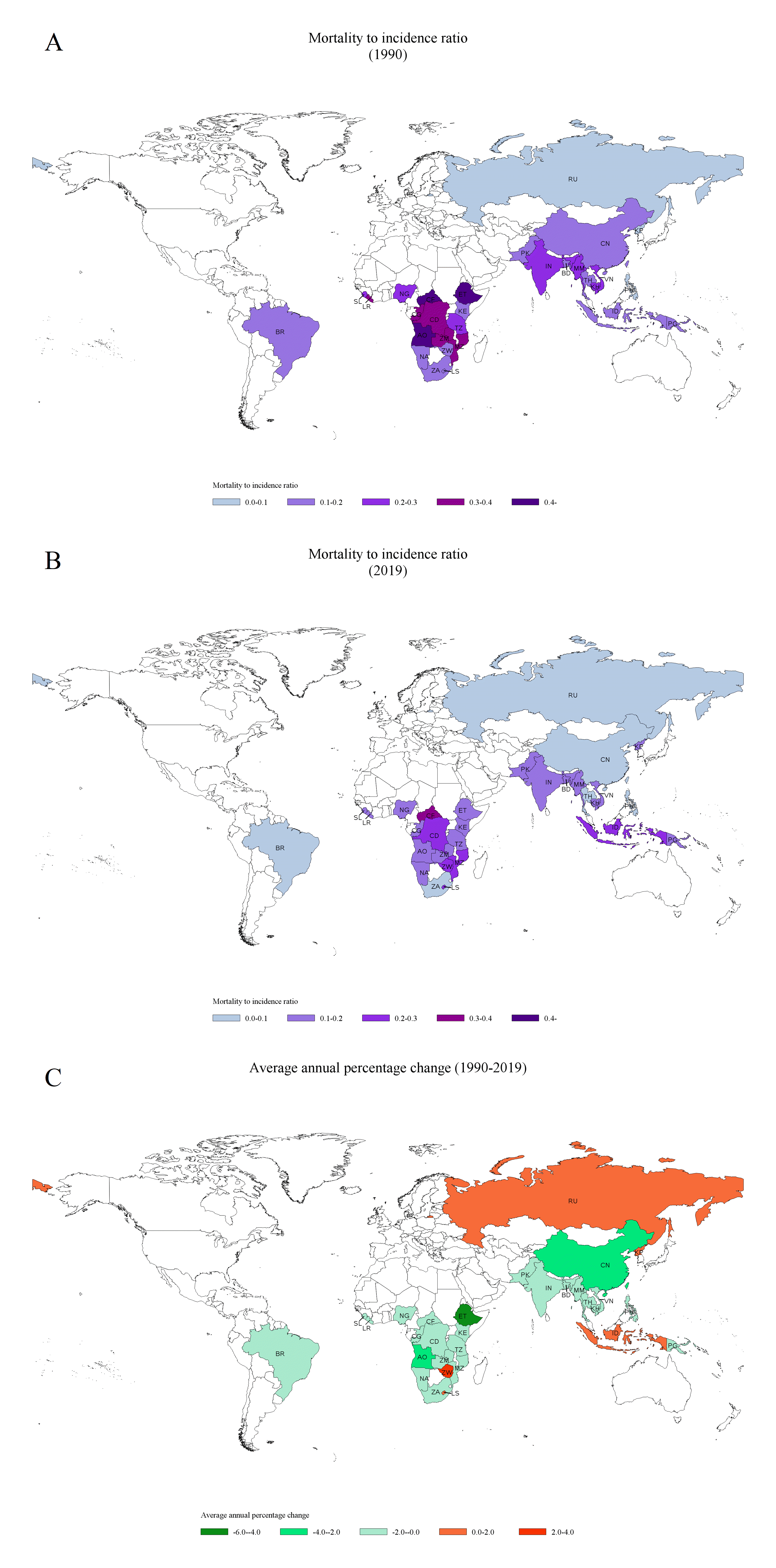

Supplement: Supplementary file 3 [file Image_3.TIF]
