# Supplementary material for: Epidemic Trends in High Tuberculosis Burden Countries During the Last Three Decades and Feasibility of Achieving the Global Targets at the Country Level
Source: Front Med (Lausanne). 2022 Mar 3;9:798465. doi: 10.3389/fmed.2022.798465 (PMC8927670; doi:10.3389/fmed.2022.798465)
Supplement: Supplementary file 7 [file Data_Sheet_1.docx]

Supplementary Material

# Supplementary Figures and Tables

## Supplementary Tables

**Supplementary Table 1.** Trajectory analysis for incidence, mortality and MI ratio of tuberculosis in the 30 HBCs

| Indicator | Group | Parameter | Estimate | Standard  Error | P value | Countries, n (%) |
| --- | --- | --- | --- | --- | --- | --- |
| Incidence | 1 | Intercept | 1704.6843 | 1085.6699 | 0.1167 | Brazil; China, 2(6.67) |
|  |  | Linear | -0.82223 | 0.54161 | 0.1293 |  |
|  | 2 | Intercept | 7718.9674 | 577.18059 | <0.0001 | Sierra Leone; Bangladesh; Kenya; Indonesia; Congo; Nigeria; Philippines, 7(23.34) |
|  |  | Linear | -3.73774 | 0.28794 | <0.0001 |  |
|  | 3 | Intercept | 4419.8761 | 627.56177 | <0.0001 | Liberia; DPR Korea; Thailand; Papua New Guinea; Viet Nam; Russian Federation, 6(19.99) |
|  |  | Linear | -2.12732 | 0.31307 | <0.0001 |  |
|  | 4 | Intercept | 2255.2415 | 1090.4991 | 0.0389 | DR Congo; Zimbabwe, 2(6.67) |
|  |  | Linear | -0.97715 | 0.54403 | 0.0728 |  |
|  | 5 | Intercept | 3166.7471 | 686.42497 | <0.0001 | Central African Republic; Lesotho; Cambodia; Namibia; South Africa, 5(16.67) |
|  |  | Linear | -1.37503 | 0.34244 | 0.0001 |  |
|  | 6 | Intercept | 9390.7094 | 542.48878 | <0.0001 | Angola; Pakistan; Zambia; Mozambique; Ethiopia; Myanmar; India; UR Tanzania, 8(26.66) |
|  |  | Linear | -4.54622 | 0.27063 | <0.0001 |  |
|  |  |  |  |  |  |  |
| Mortality | 1 | Intercept | 612.99465 | 197.5689 | 0.0020 | DPR Korea; Thailand; Papua New Guinea; Brazil; China; Russian Federation, 6(20) |
|  |  | Linear | -0.29955 | 0.09856 | 0.0024 |  |
|  | 2 | Intercept | 2638.8239 | 153.03517 | <0.0001 | Liberia; Sierra Leone; Pakistan; Bangladesh; Kenya; Indonesia; Nigeria; Viet Nam; Philippines; India, 10(33.33) |
|  |  | Linear | -1.2952 | 0.07635 | <0.0001 |  |
|  | 3 | Intercept | 2328.9878 | 182.907 | <0.0001 | Cambodia; Namibia; Zimbabwe; Congo; Myanmar; UR Tanzania; South Africa, 7(23.33) |
|  |  | Linear | -1.13121 | 0.09125 | <0.0001 |  |
|  | 4 | Intercept | -5391.333 | 483.94296 | <0.0001 | Lesotho, 1(3.33) |
|  |  | Linear | 2.75043 | 0.24143 | <0.0001 |  |
|  | 5 | Intercept | 6061.4963 | 216.42574 | <0.0001 | DR Congo; Angola; Zambia; Mozambique; Ethiopia, 5(16.67) |
|  |  | Linear | -2.98 | 0.10797 | <0.0001 |  |
|  | 6 | Intercept | 2394.9545 | 483.94276 | <0.0001 | Central African Republic, 1(3.33) |
|  |  | Linear | -1.10464 | 0.24143 | <0.0001 |  |
|  |  |  |  |  |  |  |
| MI ratio | 1 | Intercept | 2.35585 | 0.60333 | 0.0001 | DPR Korea; Thailand; Brazil; China; Russian Federation, 5(16.67) |
|  |  | Linear | -0.00113 | 0.0003 | 0.0002 |  |
|  | 2 | Intercept | -8.954 | 0.77889 | <0.0001 | Lesotho; Indonesia; Zimbabwe, 3(10) |
|  |  | Linear | 0.00459 | 0.00039 | <0.0001 |  |
|  | 3 | Intercept | 6.00212 | 0.34833 | <0.0001 | Liberia; Sierra Leone; Papua New Guinea; Pakistan; Cambodia; Bangladesh; Namibia; Kenya; Nigeria; Viet Nam; Myanmar; Philippines; India; UR Tanzania; South Africa, 15(50) |
|  |  | Linear | -0.00291 | 0.00017 | <0.0001 |  |
|  | 4 | Intercept | 17.98159 | 0.60333 | <0.0001 | DR Congo; Angola; Zambia; Ethiopia; Congo, 5(16.67) |
|  |  | Linear | -0.00882 | 0.0003 | <0.0001 |  |
|  | 5 | Intercept | 4.56957 | 0.95394 | <0.0001 | Central African Republic; Mozambique, 2(6.67) |
|  |  | Linear | -0.00209 | 0.00048 | <0.0001 |  |

MI ratio: mortality to incidence ratio; HBC: high burden country; DR Congo: Democratic Republic of the Congo; DPR Korea: Democratic People's Republic of Korea; UR Tanzania: United Republic of Tanzania.

## Supplementary Figures

## Supplementary Figure 1. Incidence, mortality and age-standardized rates of tuberculosis in the 30 HBCs, 1990-2019.

HBC: high burden country; AAPC: average annual percent change; *: significant at 0.05 level.

**Supplementary Figure 2.** Mortality of tuberculosis in the 30 HBCs and its average annual percent change: (A) Mortality rate in 1990, (B) Mortality rate in 2019, (C) Average annual percent change of mortality rate from 1990 to 2019.

HBC: high burden country; AO: Angola; BD: Bangladesh; BR: Brazil; KH: Cambodia; CF: Central African Republic; CN: China; CG: Congo; KP: Democratic People's Republic of Korea; CD: Democratic Republic of the Congo; ET: Ethiopia; IN: India; ID: Indonesia; KE: Kenya; LS: Lesotho; LR: Liberia; MZ: Mozambique; MM: Myanmar; NA: Namibia; NG: Nigeria; PK: Pakistan; PG: Papua New Guinea; PH: Philippines; RU: Russian Federation; SL: Sierra Leone; ZA: South Africa; TH: Thailand; TZ: United Republic of Tanzania; VN: Viet Nam; ZM: Zambia; ZW: Zimbabwe.

**Supplementary Figure 3.** Mortality to incidence ratio of tuberculosis in the 30 HBCs and its average annual percent change: (A) Mortality to incidence ratio in 1990, (B) Mortality to incidence ratio in 2019, (C) Average annual percent change of mortality to incidence ratio from 1990 to 2019.

HBC: high burden country; AO: Angola; BD: Bangladesh; BR: Brazil; KH: Cambodia; CF: Central African Republic; CN: China; CG: Congo; KP: Democratic People's Republic of Korea; CD: Democratic Republic of the Congo; ET: Ethiopia; IN: India; ID: Indonesia; KE: Kenya; LS: Lesotho; LR: Liberia; MZ: Mozambique; MM: Myanmar; NA: Namibia; NG: Nigeria; PK: Pakistan; PG: Papua New Guinea; PH: Philippines; RU: Russian Federation; SL: Sierra Leone; ZA: South Africa; TH: Thailand; TZ: United Republic of Tanzania; VN: Viet Nam; ZM: Zambia; ZW: Zimbabwe.

**Supplementary Figure 4.** Incidence trends of tuberculosis by different age groups in the 30 HBCs, 1990-2019.

HBC: high burden country; AAPC: average annual percent change; *: significant at 0.05 level.

**Supplementary Figure 5.** Mortality trends of tuberculosis by different age groups in the 30 HBCs, 1990-2019.

HBC: high burden country.

**Supplementary Figure 6.** Average annual percent changes of mortality by different age and sex groups in the 30 HBCs, 1990-2019.

HBC: high burden country.
